# Bilateral tibial Brodie’s abscess in a young patient treated with BAG-S53P4: case report

**DOI:** 10.1186/s13052-019-0685-z

**Published:** 2019-07-26

**Authors:** Andrea Cossio, Jole Graci, Antonino Salvatore Lombardo, Marco Turati, Maria Luisa Melzi, Marco Bigoni, Giovanni Zatti

**Affiliations:** 1 Orthopedic Department, San Gerardo Hospital, University of Milano – Bicocca, Monza, Italy; 2 Department of Paediatric Orthopaedics, Saint Joseph Hospital, Marseille, France; 3 School of Medicine and Surgery, University of Milano – Bicocca, Monza, Italy; 4 Pediatrics Department, San Gerardo Hospital, University of Milan – Bicocca, Monza, Italy

**Keywords:** Brodie’s abscess, BAG-S53P4, Pediatric patient, Osteomyelitis, Bioglass

## Abstract

**Background:**

Brodie’s abscess is a form of chronic pyogenic osteomyelitis that usually affects the cancellous part of the long bones in children. Its treatment is represented by antibiotic therapy alone or in association with surgical procedures.

**Case presentation:**

A 12-years-old male affected by a Brodie’s abscess of the tibia involving the distal growth plate was admitted to the Pediatric Department for a conservative treatment. After several attempts of antibiotic therapy interrupted for intolerance manifestations he was surgically treated with bioactive glass BAG-S53P4 (BonAlive, BonAlive Biomaterials Ltd., Biolinja, Finland), with excellent results.

**Conclusions:**

In our experience BAG-S53P4 has proven to be an effective bone substitute without side effects even in the pediatric population. In our case it eradicated the infection without interfere with the growth; neither epiphysiodesis nor other disorders were found during the follow-up.

For the publication of this case report we followed the CARE guidelines for good clinical case reports; the parents gave consent for publication.

## Background

A Brodie’s abscess is chronic pyogenic osteomyelitis that usually affects the cancellous portion of the long bones in children when the virulence of the organism and the resistance of the patient are balanced [1]. The lower limb, and in particular the tibia, is more frequently affected [2].

*Staphylococcus aureus* is the causative organism in 30–60% of cases. Other organisms encountered are *Streptococcus, Pseudomonas*, *Haemophilus influenzae* and *Kingella kingae* [3]. However, in almost 25–50% of cases no organism is cultured [4].

The treatment of Brodie’s abscess varies: in children there are reports of successful treatment with antibiotics combined with immobilization or with curettage and postoperative antibiotics for 6–8 weeks. Also antibiotic-impregnated PMMA beads are available [5, 6].

Some authors reported that systemic antibiotics alone might be effective in treating primary subacute osteomyelitis in children and suggested that surgery should be reserved for aggressive lesions and those not responding to antibiotic therapy [7–9]. Prolonged pharmacological therapy may result in high antibiotic serum concentration associated with nephrotoxic and ototoxic effects and allergic complications.

The curettage of abscess cavity and filling with cancellous bone grafting has been reserved mainly for those with large cavity diameters > 3 cm and for aggressive lesions with ESR > 40 mm/hr. Histological and culture examinations are recommended to identify the responsible microorganism and set a targeted antibiotic therapy [5, 9–11].

Surgical debridement and local gentamicin-PMMA beads implantation doesn’t require prolonged parenteral antibiotic therapy, reducing hospital stay, medical costs and complications. At the same time it is not free of drawbacks: it requires a second intervention for the beads removal [5] and it can cause allergic reactions and bacterial antibiotic resistance.

An alternative treatment recently introduced against osteomyelitis is represented by BAG-S53P4: an osteoconductive bone substitute with proven antibacterial, proangiogenic and bone bonding properties. It’s employed in the orthopaedic field as a bone filler in the treatment of osteomyelitis, benign bone tumors and open fractures [12].

Its orthopaedic application in adulthood is increasing while its use in pediatric patients is limited. Its efficacy has been proven in children affected by recurrent aneurysmal bone cyst [13], intra-articular open fracture [14] and benign bone tumors [15, 16].

We report the case of a 12 years old male surgically treated with BAG-S53P4 for a Brodie’s abscess of the left tibia involving the distal growth plate.

## Case presentation

In June 2016 a 12-years-old boy was evaluated for pain and swelling of the left ankle exacerbated by sport, associated with limp and hyperpyrexia (T max 39 °C) and responsive to paracetamol.

**Fig. 1:**
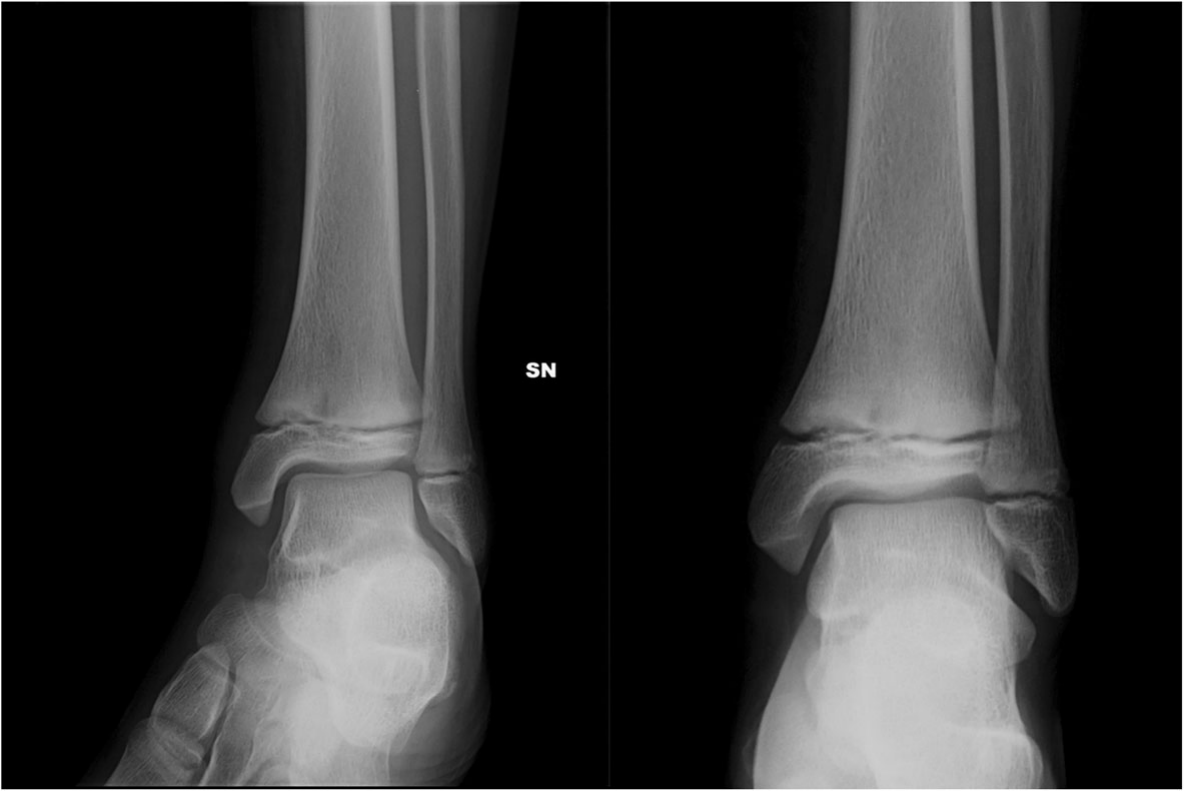
Antero-posterior and lateral view radiographs of the left tibia showing an irregularity of the nucleus of ossification suspected for Brodie’s abscess

**Fig. 2:**
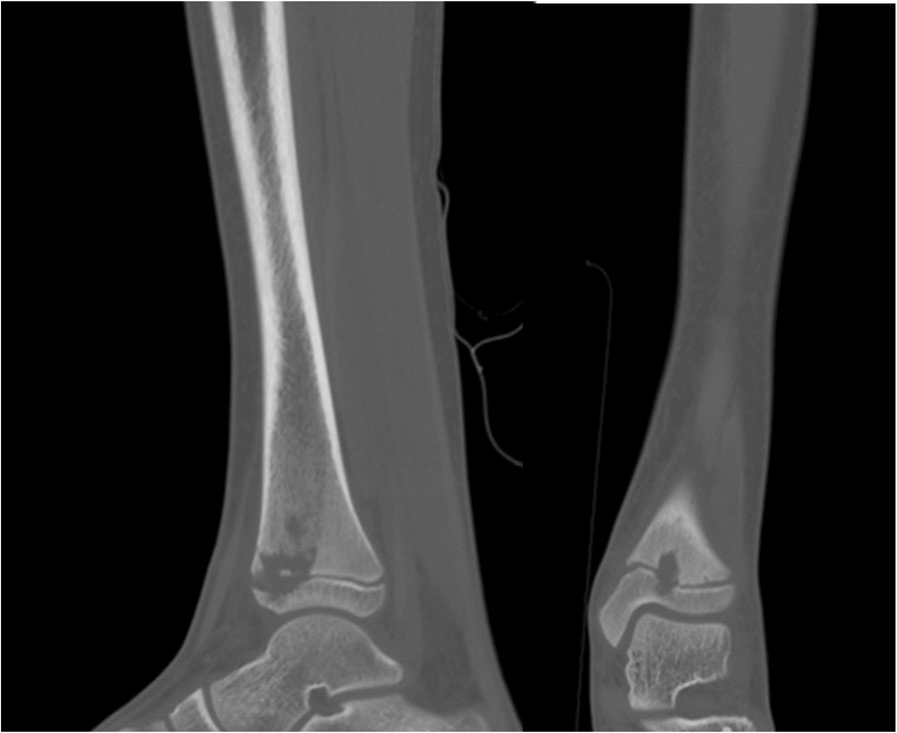
Antero-posterior and lateral view CT scan of the left tibia showing a cavity that involves the growth plate with sclerotic edge in absence of periosteal reaction; this radiological finding confirms the suspicion of Brodie’s abscess

A radiographic study (Fig. 1) showed an irregularity of the nucleus of ossification of the left distal tibia. The subsequent MRI showed a distal metaphyseal bone lesion of 23 mm diameter with surrounding oedema of the distal diaphysis of the tibia, the distal growth plate and the joint surface. The CT scan (Fig. 2) identified a cavity involving the growth plate with sclerotic edge in absence of periosteal reaction; a contralateral CT scan showed a subcentimetric cavity with sclerotic margins localized in the distal metaphysis of the right tibia just above the growth plate.

The patient was admitted to the Pediatric Department for appropriate treatment and in-depth diagnostic investigations with a suspicion of Brodie’s abscess of the left tibia; a subclinical lesion was also present on the right tibia and needed monitoring.

He underwent CT guided biopsy of the lesion for culture examination and a wash of the cavity with physiological solution was performed. The sample was positive for methicillin-sensitive *S. aureus*; the organism was sensitive to levofloxacin (M.I.C. <= 0.12) and oxacillin (M.I.C. 0.5), so a proper antibiotic therapy was carried out (oral levofloxacin and i.v. oxacillin).

Immobilization with cast without weight-bearing on the left leg was recommended for 5 weeks; the patient monitoring included blood tests (CRP value at the admission: 0.18 mg/dL).

After 20 days of antibiotic therapy the patient showed skin rash, hyperpyrexia (T max 39 °C), raising of the inflammation indices (CRP 4.7 mg/dL, PCT 1.0 ng/mL), leukopenia and thrombocytopenia. Blood cultures resulted negative, so the antibiotic therapy was stopped with the suspicion of adverse effects to oxacillin; intravenous hydration, corticosteroids and antihistamines were administered with progressive disappearance of the picture of adverse effects.

Twenty eight days after the biopsy a radiography of the left leg was performed: the lesion appeared stable with no signs of cavity collapse; given the absence of radiographic and local clinical signs of worsening, gradual weight-bearing was permitted without cast.

Forty days after the biopsy a new oral therapy was prescribed (rifampicin + levofloxacin). Immediately after levofloxacin administration the patient showed skin rash, headache and vomit, so the drug was stopped.

Forty three days after the biopsy, given the remission of the allergic manifestations due to levofloxacin suspension, rifampicin and trimethoprim + sulfamethoxazole were introduced.

Blood tests showed leukopenia, therefore the antibiotic therapy was stopped again and abundant intravenous hydration was administered. After the normalization of blood exams, rifampicin and doxycycline oral therapy for 6 weeks was prescribed.

Two months after the biopsy a MRI with contrast of the left ankle showed a reduction of osseous oedema and an enhancement of the lesion across the growth plate. Considering the difficulties encountered during the antibiotic therapy and the MRI evolution of the lesion, a surgical approach was planned in order to eradicate the infection.

**Fig. 3:**
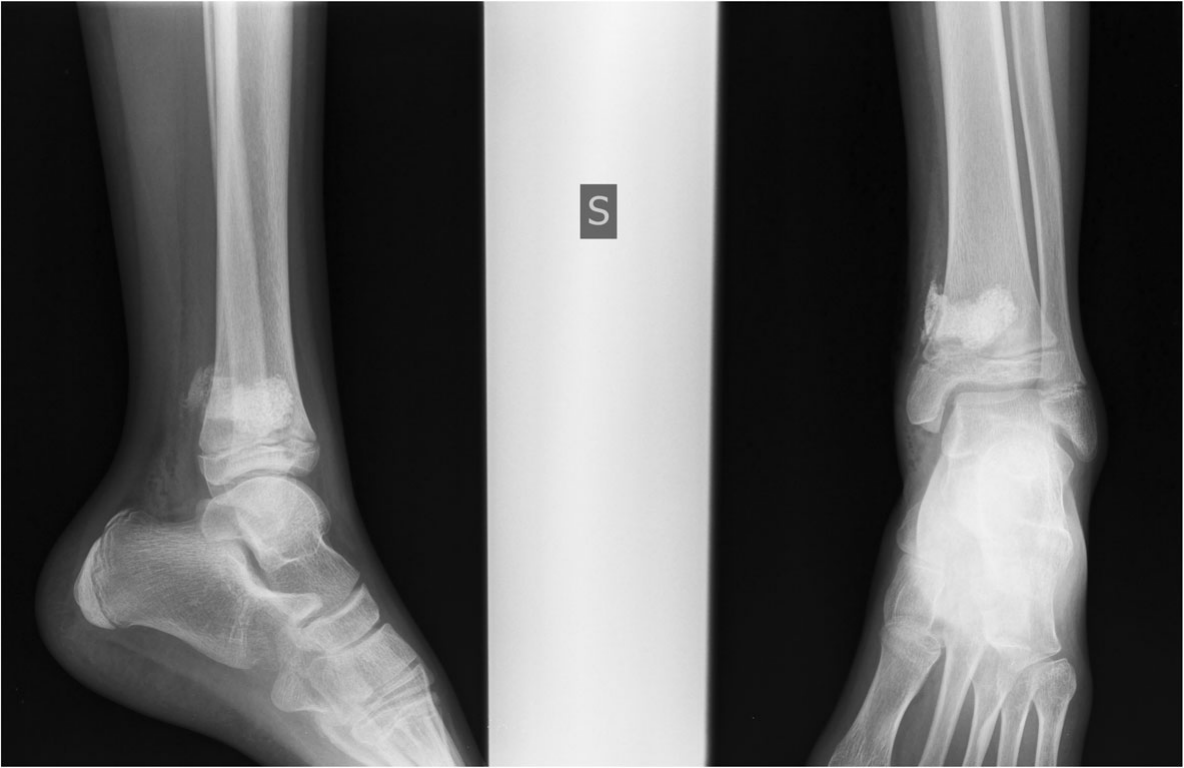
Antero-posterior and lateral view postoperative radiographs of the left tibia showing filling of the bone defect with BAG-S53P4 granules. Traces of spilled material can be observed in soft tissues

The surgical procedure consisted of curettage and removal of the infected bone, sample cavitary bone collection for histological and bacteriological examination, washing with physiological solution and proper filling of the bone defect with BAG-S53P4 granules (Fig. 3).

The culture examination of the intraoperative sample was negative and histological exam showed signs of chronic inflammation.

Postoperative policy entailed using walking aids without weight-bearing on the left limb and physio-kinesiotherapy for 1 month.

**Fig. 4:**
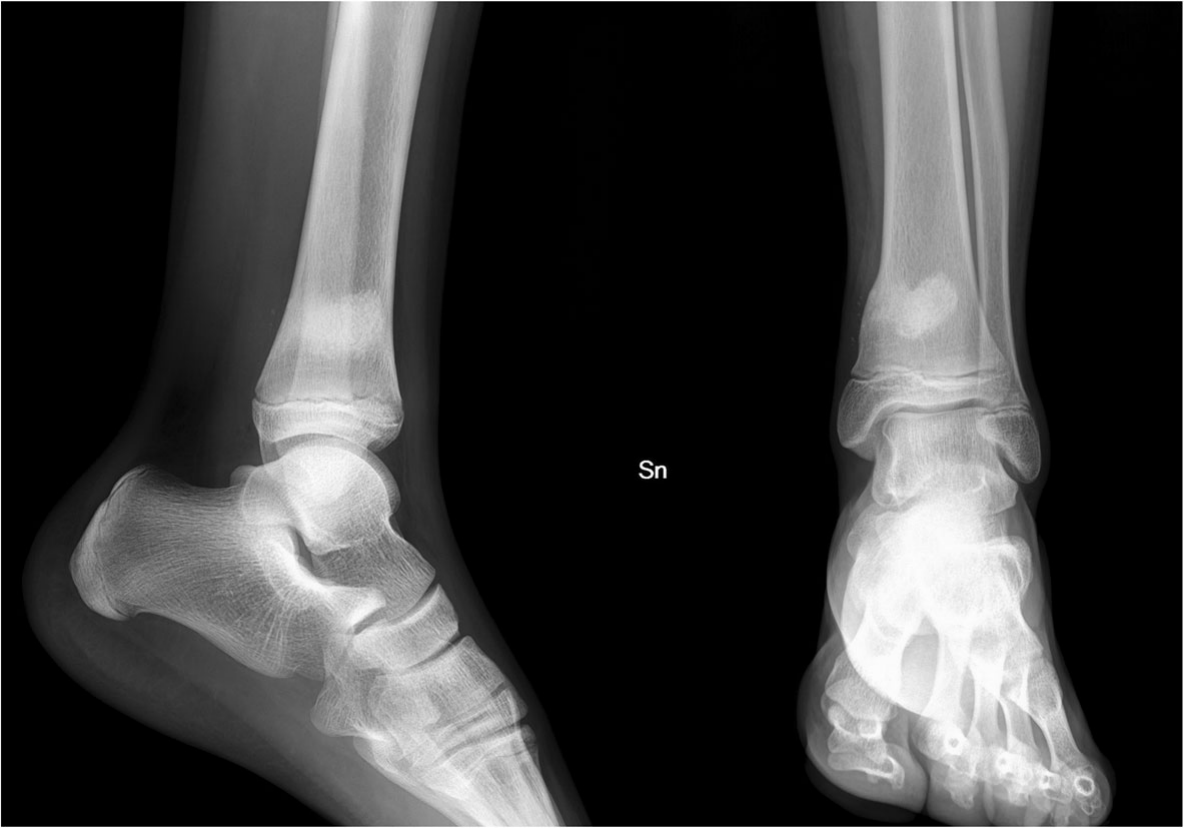
Antero-posterior and lateral view 17-months postoperative radiographs of the left tibia showing good bone filling of the neocavity without alterations of the growth plate. Granules spilled in soft tissues have been reabsorbed and are not radiographically visible

Clinical and radiographic evaluations were performed for both ankles at 1, 2, 4, 12 months after surgery. The clinical observation at the last follow-up (17 months) showed no signs of reactions to the biomaterial, no clinical or haematological indices for infection recurrence and radiographic examinations showed good bone filling of the neocavity without alterations of the growth plate (Fig. 4). In addition, there were no clinical or radiological signs of recurrence of the Brodie’s abscess in the contralateral tibia.

## Discussion and conclusions

In our case, during the pharmacological treatment, the patient developed hypersensitivity and adverse reactions to the multiple antibiotics administered, so it was necessary to stop the therapy and to treat the unexpected complications. In this context it was helpful to attempt an effective surgical treatment without the use of standard antibiotics, therefore we chose an alternative antibacterial agent represented by BAG-S53P4.

BAG-S53P4 antibacterial properties depend on the increase of the local pH and osmotic pressure through the release of sodium and calcium ions and phosphorus salts, which inhibits the bacterial adhesion and proliferation [17]. It exerts its local bactericidal action without the addition of local antibiotic, so no adverse reactions and induction of bacterial resistance to antibiotics are known.

Recently various studies have been published about the use of BAG-S53P4 in the treatment of chronic osteomyelitis in adults. Lindfors et al [18] reported a success rate of 90.9% (10/11) in controlling bone infection with a mean follow-up of 24 months.

Geurts et al [19] reported excellent clinical and radiological results in all fifteen patient treated with debridement of the osteomyelitic lesion and filling with BAG-S53P4. One pediatric patient was treated in this study: a 14-years-old female affected by an haematogenous chronic osteomyelitis of the tibia. Over a follow-up of 20.2 months she showed no signs of reinfection.

Lastly Lindfors et al [20] in their multinational study reported a success rate of 90% against chronic osteomyelitis in one hundred and sixteen patients with a median age of 48 years (range 15–87 years).

In our experience BAG-S53P4 has proven to be an effective bone substitute without side effects even in the pediatric field. The leakage of BAG particles from the bone cavity in which they were placed didn’t result in complications or heterotopic calcifications; in fact traces of spilled material, radiographically visible in the early stages, underwent progressive resorption during the follow-up.

In our case, the Brodie’s abscess involved the growth plate of the distal tibia: despite the curettage of the infected bone and the contact between the physis and the bioglass used to fill the bone cavity, the BAG-S53P4 didn’t interfere with the growth. Neither epiphysiodesis nor other disorders were found during the follow-up.

At 17 months from surgical treatment our patient is still free of infection.

To date the literature is lacking in data about the application of BAG-S53P4 in the treatment of pediatric Brodie’s abscess.

In our case the use of BAG-S53P4 was imposed by the necessity of an effective surgical treatment without the use of antibiotic therapy; after the treatment our patient didn’t show any complication and at present he exhibits a complete *restitutio ad integrum*.

Further studies and long-term follow-up need to be performed in order to determine whether BAG-S53P4 could be a suitable and safe bone substitute in the treatment of pediatric population.

## Data Availability

Not applicable.

## Abbreviations

BAG-S53P4: Bioactive glass-S53P4
CRP: C Reactive Protein
CT: Computed tomography
ESR: Erythrocyte sedimentation rate
i.v: Intravenous
M.I.C: Minimum inhibitory concentration
MRI: Magnetic Resonance Imaging
PMMA: Polymethyl methacrylate
